# Pleurotomy with subxyphoid pleural drain affords similar effects to pleural integrity in pulmonary function after off-pump coronary artery bypass graft

**DOI:** 10.1186/1749-8090-7-11

**Published:** 2012-01-25

**Authors:** Solange Guizilini, Douglas W Bolzan, Sonia M Faresin, Raquel F Ferraz, Kelly Tavolaro, Andrea A Cancio, Walter J Gomes

**Affiliations:** 1 Department of Medicine, Cardiology Discipline, Pirajussara and São Paulo Hospitals, Escola Paulista de Medicina, Federal University of Sao Paulo, (Napoleao de Barros, 715), Sao Paulo, (04024-002), Brazil; 2 Department of Human Movement Sciences, Physiotherapy School, Federal University of Sao Paulo, (Ana Costa, 95), Santos, (11060-001), Brazil; 3 Department of Medicine, Pneumology Discipline, São Paulo Hospital, Escola Paulista de Medicina, Federal University of Sao Paulo, (Pedro de Toledo, 720), Sao Paulo, (04039-002), Brazil; 4 Department of Medicine, Cardiovascular Surgery Discipline, Pirajussara and São Paulo Hospitals, Escola Paulista de Medicina, Federal University of Sao Paulo, (Napoleao de Barros, 715), Sao Paulo, (04024-002), Brazil

**Keywords:** Off pump coronary artery bypass grafting, Internal thoracic artery, Pleurotomy, Pulmonary function, Pleural drain, Pain.

## Abstract

**Background:**

Exacerbation of pulmonary dysfunction has been reported in patients receiving a pleural drain inserted through the intercostal space in comparison to patients with an intact pleura undergoing coronary artery bypass grafting (CABG). Evidence suggests that shifting the site of pleural drain insertion to the subxyphoid position minimizes chest wall trauma and preserves respiratory function in the early postoperative period. The aim of this study was to compare the pulmonary function parameters, clinical outcomes, and pain score between patients undergoing pleurotomy with pleural drain placed in the subxyphoid position and patients with intact pleural cavity after off-pump CABG (OPCAB) using left internal thoracic artery (LITA).

**Methods:**

Seventy-one patients were allocated into two groups: I (n = 38 open left pleural cavity and pleural drain inserted in the subxyphoid position); II (n = 33 intact pleural cavity). Pulmonary function tests and clinical parameters were recorded preoperatively and on postoperative days (POD) 1, 3 and 5. Arterial blood gas analysis and shunt fraction were evaluated preoperatively and in POD1. Pain score was assessed on POD1. To monitor pleural effusion and atelectasis chest radiography was performed routinely 1 day before operation and until POD5.

**Results:**

In both groups a significant impairment was found in lung function parameters until on POD5. However, no significant difference in forced vital capacity and forced expiratory volume in 1 second were seen between groups. A significant decrease in partial pressure of arterial oxygen and an increase in shunt fraction values were observed on POD1 in both groups, but no statistical difference was found when the groups were compared. Pleural effusion and atelectasis until on POD5 were similar in both groups. There were no statistical differences in pain score, duration of mechanical ventilation and postoperative hospital stay between groups.

**Conclusion:**

Subxyphoid insertion of pleural drain provides similar effects to preserved pleural integrity in pulmonary function, clinical outcomes, and thoracic pain after OPCAB. Therefore, our results support the hypothesis that once pleural cavities are incidentally or purposely opened during LITA dissection, subxyphoid placement of the pleural drain is recommended.

## Background

Coronary artery bypass grafting (CABG) using the left internal thoracic artery (LITA) grafts remains the gold standard for treatment of patients with coronary disease and has been associated with higher survival and better quality of life [1]. Despite these long-terms benefits, LITA harvesting has been demonstrated to induce changes in pulmonary mechanical behavior and lead to a greater impairment to pulmonary function in the early postoperative period and an increased risk of respiratory complications [2,3]. This harm is significantly attributed to the pleural cavity opening and need of chest tube placement through intercostal space [4-6].

Exacerbation of pulmonary dysfunction has been reported in patients receiving a pleural drain inserted through the intercostal space in comparison to patients with an intact pleura undergoing off-pump CABG (OPCAB) [7]. Additionally, contemporary evidence suggests that shifting the site of pleural drain insertion to the subxyphoid position minimizes chest wall trauma and preserves respiratory function in the early postoperative period [8,9].

The aim of this study was to compare the pulmonary function parameters, clinical outcomes, and pain score between patients undergoing pleurotomy with pleural drain placed in the subxyphoid position and patients with an intact pleural cavity after OPCAB using LITA.

## Methods

### Patient selection

Two hundred fifty-eight patients scheduled for CABG were screened for inclusion in this study. Seventy-one patients undergoing elective first-time OPCAB fulfilled the inclusion criteria and were in fact analyzed. The following exclusion criteria were used: preoperative (emergency surgery, chronic obstructive pulmonary disease, skeletal abnormalities causing pulmonary restriction, previous cardiac surgery, incapable of performing spirometry, unstable angina, refusal to participate in the study, and ejection fraction < 50%), intraoperative (conversion to cardiopulmonary bypass, bilateral opening of pleural cavities, and protocol violation), postoperative (death, early hospital discharge, stroke, and prolonged hemodynamic instability).

The 71 patients included in this study were allocated into two groups: group I (38 patients with incidental opening of the left pleural cavity and pleural drain inserted in the subxyphoid position); and group II (33 patients with intact pleural cavity). The Human Ethics Committee of the Federal University of São Paulo approved this study, and each patient gave informed written consent before surgery.

### Pulmonary Function Assessment

Forced vital capacity (FVC) and forced expiratory volume in 1 second (FEV_1_) were evaluated bedside using a portable spirometer (Spirobank G, MIR, Rome, Italy), according to the standards of the American Thoracic Society [10]. Each test was repeated at three times, and the best result was selected. Pulmonary function testing was performed preoperatively and on postoperative days 1, 3, and 5 (POD1, POD3, POD5) by the same respiratory physiotherapist.

Arterial blood gas measurements (partial pressure of arterial oxygen [PaO_2_] and partial pressure of carbon dioxide [PaCO_2_]) and pulmonary shunt fraction were determined in preoperative and POD1 with patient breathing room air, always before performing spirometry. Pulmonary shunt fraction was calculated using software Oxygen Status Algorithm, version 2.0; Mads&Ole Siggaard; Radiometer. This program needs the arterial blood gas and the fraction of inspired oxygen to calculate the percentage of blood that is not supplied by oxygen.

### Anesthesia and ventilation management

All patients received a standard anesthetic technique, induction with etomidate and midazolam, maintenance with sufentanil and isofluorane (0.5% to 1%) and were mechanically ventilated with a tidal volume of 8 ml/kg, and a respiratory rate adapted to maintain normocapnia, with a fraction of inspired oxygen of 50% without positive end-expiratory pressure. Intraoperative fluids were given according to the discretion of the anesthetist.

### Surgical procedures

The OPCAB surgery was performed through a median sternotomy, using LITA complemented with additional saphenous vein grafts. The LITA was harvested in a skeletonized fashion, isolating it from the fascia, the veins, and adipose tissue. Skeletonization was performed from the origin down to the bifurcation, and side branches were ligated with small-sized hemostatic clips only at the LITA side. Meticulous care is routinely taken to preserve the integrity of the pleura during left internal thoracic artery harvesting. In all cases in which the pleural cavity was incidentally opened, a soft tubular and curved pleural drain (¼ inch) was inserted, positioned in the left costophrenic sinus and exteriorized at the subxyphoid region. In all patients, a straight mediastinal drain was also placed via a subxyphoid approach. A heated water mattress was used to keep all patients normothermic throughout the operation.

The OPCAB surgery has followed our protocol [11]. Briefly, with systemic heparinization to achieve an activated clotting time exceeding 250 seconds, occlusion of the coronary artery was accomplished by using a proximal soft silicone snare. The distal anastomosis was performed with a 7-0 polypropylene running suture. The vein top ends were then attached to the ascending aorta using side-bite clamping. An Octopus 3 (Medtronic, Inc, Minneapolis, MN) suction stabilizer was used in all cases.

### Postoperative Management

At the end of surgery, all patients were transferred to the surgery cardiac intensive care unit. Mechanical ventilation was started using synchronized intermittent mandatory ventilation with tidal volume of 8 ml/Kg, pressure support to maintain this volume, rate of 12 to 14 breaths/min, an inspiratory/expiratory ratio of 1:2, positive end-expiratory pressure of 5 cmH_2_O, and fraction of inspired oxygen to keep arterial oxygen saturation above 90%. Weaning from ventilation was performed when the patient was hemodynamically stable and sufficiently alert to maintain spontaneous ventilation and acceptable blood gas values. All patients had invasive continuous monitoring of arterial blood pressure and central venous pressure, heart rhythm and pulse oximetry. All patients received the same analgesic protocol administered during the POD5. Pain sensation was assessed on POD1 before spirometry and quantified by a modified standard score (0 = no pain to 10 = unbearable pain) [12].

The length of mechanical ventilation and postoperative hospital stay, pleural effusion, and atelectasis were recorded. Chest roentgenograms; taken preoperatively and on the POD1, POD3 and POD5; were evaluated by a radiologist who was blind to the patient's groups. Pleural effusion was considered relevant when exceeding the phrenocostal angle and fluid drainage was monitored hourly. The drains (mediastinal and/or pleural) were routinely removed on POD2. Atelectasis was acknowledged when a clear radiologic shadow exceeded 15 mm in width, with linear atelectasis being disregarded in this study.

During the postoperative phase, the patients were encouraged to carry out their breathing exercises under the guidance of the physiotherapist until discharge.

### Statistical analysis

Statistical analysis was performed using GraphPad Prism 3.0 Software (GraphPad Software Inc, San Diego, CA). Data are reported as mean ± standard deviation. The FVC and FEV_1 _values were expressed as a percentage of the preoperative value. Within-group variables comparing preoperative versus postoperative values were evaluated using paired Student's t tests and ANOVA for repeated measures. Normally distributed continuous variables were compared between groups using unpaired Student's t tests and abnormally distributed data were compared using Mann-Whitney test. The categorical variables were analyzed using the Pearson Chi-square test. A *p *value of the less than 0.05 was considered statistically significant.

## Results

**Figure 1:**
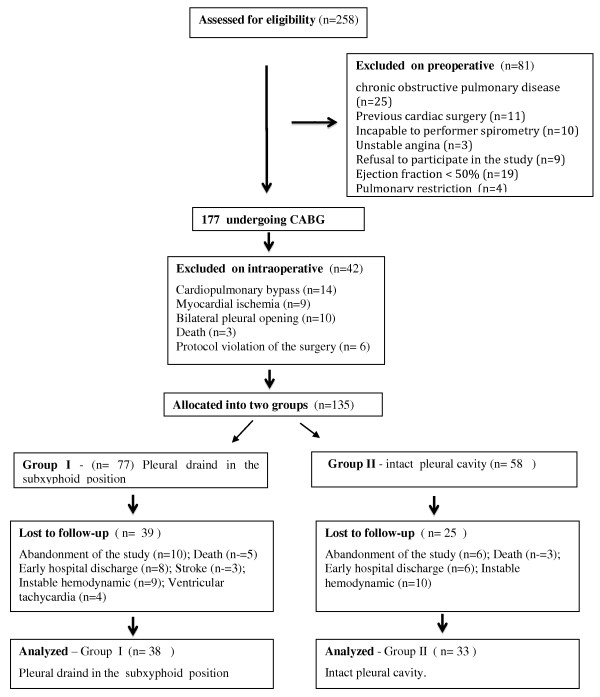
**Participants flow**.

During the study period, 258 patients fulfilled eligibility criteria. From that sample, 71 were in fact analyzed (Figure 1). Preoperative and intraoperative patient characteristics are summarized in table 1.

**Table 1:** Pre and intraoperative clinical and demographic parameters.

Variables	Group I (n = 38)	Group II (n = 33)	p Value
Age (years)	59.84 ± 8.44	60.18 ± 7.29	0.85
Male/female	30/08	27/6	0.76
BMI (kg/m^2^)	26.96 ± 3.34	27.58 ± 3.40	0.44
Pulmonary function			
FVC (L)	3.55 ± 0.61	3.64 ± 0.64	0.27
% predicted	97.72 ± 13.42	99.44 ± 13.07	0.29
FEV_1 _(L)	2.78 ± 0.44	2.86 ± 0.49	0.23
% predicted	100.6 ± 12.20	98.88 ± 11.25	0.26
PaO_2 _(mmHg)	76.18 ± 5.75	77.36 ± 4.49	0.34
PaCO_2 _(mmHg)	38.47 ± 3.47	39.15 ± 3.65	0.42
Shunt fraction (%)	0.04 ± 0.02	0.03 ± 0.02	0.09
Operative time (min)	304.7 ± 20.70	300.6 ± 19.75	0.39
Grafts per patient	2.47 ± 0.68	2.54 ± 0.66	0.65

Data are shown as mean ± standard deviation. (BMI = body mass index; FEV_1 _= Forced expiratory volume in 1 second; FVC = forced vital capacity; PaCO_2 _= partial pressure of arterial carbon dioxide; PaO_2 _= partial pressure of arterial oxygen).

No statistical difference was found in terms of age, gender, body mass index, spirometric parameters, arterial blood gas analysis, operative time and number of grafts per patient between groups.

Postoperatively, in both groups there was a significant impairment in lung function parameters until POD5 (p < 0.001). However, no significant difference in FVC and FEV_1 _(expressed as a percentage of the preoperative values) was seen between the two groups (Table 2).

**Table 2:** Pulmonary function test values on the POD 1, 3 and 5, in percentage of the preoperative values.

	POD1			POD3			POD5		
**Variables**	**Group I**	**Group II**	**p**	**Group I**	**Group II**	**p**	**Group I**	**Group II**	**p**
FVC (%)	49.04 ± 10.78	52.08 ± 10.86	0.12	61.20 ± 11.82	64.10 ± 11.11	0.14	75.14 ± 14.67	78.79 ± 11.28	0.12
FEV_1_(%)	46.65 ± 9.54	48.72 ± 9.24	0.18	65.05 ± 10.40	62.63 ± 10.93	0.17	78.26 ± 13.93	80.03 ± 11.40	0.28

p values refer to the difference of the FVC and FEV_1 _between groups. Data are shown as mean ± standard deviation. (FEV_1 _= Forced expiratory volume in 1 second; FVC = forced vital capacity; POD = postoperative day).

A significant decrease in PaO_2 _and an increase in pulmonary shunt fraction values were observed on POD1 in both groups (p < 0.05), but no statistical difference was found when the groups were compared (Table 3).

**Table 3:** Arterial blood gas and shunt fraction measurements in the POD1.

	Group I	Group II	p value
PaO_2 _(mmHg)	61.95 ± 6.71	63.61 ± 6.59	0.29
PaCO_2 _(mmHg)	43.05 ± 6.27	41.03 ± 5.73	0.16
Shunt fraction (%)	0.21 ± 0.07	0.19 ± 0.06	0.18

Data are shown as mean ± standard deviation. (PaCO_2 _= partial pressure of arterial carbon dioxide; PaO_2 _= partial pressure of arterial oxygen; POD1 = postoperative day 1).

The incidence of pleural effusion and atelectasis until POD5 were similar in both groups (Table 4).

**Table 4:** Postoperative clinical variables

Variables	Group I (n = 38)	Group II (n = 33)	p value
Atelectasis, n (%)	9 (23)	7(21)	0.80
Pleural effusion, n (%)	6 (16)	5 (15)	0.47
Pain score *	5.68 ± 1.25	5.54 ± 1.06	0.22
Mechanical ventilation time, hours*	9.84 ± 1.53	9.24 ± 1.20	0.07
Hospital stay, days*	6.57 ± 1.30	6.14 ± 1.34	0.23

* Data are shown as mean ± standard deviation.

None of the patients with open left pleural cavity and subxyphoid position pleural drain presented drain malfunction, or needed further thoracentesis.

Similar results were found when pain score, mechanical ventilation duration, and postoperative hospital stay were compared between groups (Table 4).

## Discussion

The present study demonstrates a significant reduction of pulmonary function in the early postoperative period of OPCAB surgery, regardless of the technique employed. There is clear evidence that postoperative pulmonary dysfunction of varying degrees is an inevitable consequence of cardiac surgery [2,3,13]. A number of factors appear to contribute to pulmonary function abnormalities including general anesthesia, median sternotomy, surgical injury and cardiopulmonary bypass [3,14]. General anesthesia combined with prolonged supine positioning results in an upward displacement of the diaphragm, and a shift in blood volume to the abdomen from the thorax, with consequent ventilation-perfusion mismatch [14]. Sternotomy itself may decrease chest wall compliance and interfere with sternal stability [2,3]. The cardiopulmonary bypass additionally causes lung injury and a delay in pulmonary recovery [15-17]. Lung injury has been largely attributed to higher degree of acute systemic and pulmonary inflammatory response [17,18].

Internal thoracic artery (ITA) has become the conduit of choice for myocardial revascularization because of its superior patency rate, reduced cardiac events and increased long-term survival compared with saphenous vein grafting [1]. However, reports have demonstrated that ITA harvesting during a CABG is an adjunctive factor for further impairment of postoperative pulmonary function, and may increase the risk of pleuropulmonary complications [2,3,13]. Previous reports attributed the postoperative poor lung function to pleurotomy followed by chest tube insertion and additional trauma to the chest wall during dissection of ITA graft [2,5,19,20].

Gullu et al [6] recommended averting routine pleura opening, as preservation of pleural integrity during ITA harvesting had positive beneficial effect on pulmonary function, pain decrease and lowered the rate of postoperative pleural effusions and atelectasis.

We have previously shown that even avoiding the systemic inflammatory response syndrome and pulmonary injury associated to cardiopulmonary bypass (i.e., OPCAB surgery), patients undergoing pleurotomy and intercostal space drain placement presented a larger decrease of the FVC and FEV_1_, oxygenation, and gas exchange in comparison to patients with intact pleura in the early postoperative period [7].

Additionally, pleural integrity is reportedly associated with other further advantages, such as a less incidence of pleuropulmonary alteration [4,5,21], reduced orotracheal intubation time [7,19,20] and lower pain score [5-7]. These findings associated to the enhanced preservation of the pulmonary function appear to be, at least in part, responsible for the shorter hospital stay found in patients with intact pleura [7,19,20].

Greater pulmonary dysfunction, increased rates of atelectasis and pleural effusion in patients undergoing to pleurotomy with an intercostal pleural drain reflects the greater degree of chest wall trauma [5,7,20,22]. Typically, pleural tube insertion through the intercostals space is associated with significant patient discomfort in the early postoperative period [22]. The friction of the drain between the ribs leads to painful sensations caused by costal periosteum and parietal pleura irritation [23,24]. The patient responds with chest wall immobilization and superficial respiratory movements, restricting deep breaths until drain removal, intensifying pulmonary capacities and volumes decreases [7,22]. All of these factors lead to a negative impact on mobilization and pulmonary toilet and increase the risk of respiratory complications in the postoperative period [4,22-24].

Evidence indicates that the degree of pulmonary dysfunction in the presence of pleurotomy depends on the insertion site of the pleural drain. Hagl et al [8] showed that subxyphoid insertion of the pleural drain leads to a significantly lower impairment of pulmonary function and less pain than intercostal insertion in the early postoperative period, similar results were demonstrated in patients undergoing OPCAB [9].

Technical questions concerning the pleural opening with subxyphoid chest drain insertion, compared to the maintenance of pleural integrity and its influence on postoperative pulmonary function have not been addressed to date. Therefore the main question is: "Does subxyphoid insertion of the pleura drain generate similar effects on pulmonary function when compared to the maintenance of pleural integrity?".

In the present study, no significant differences on pulmonary function parameters (FVC and FEV_1_), oxygenation and shunt fraction between the two groups were found during the early postoperative period. Our study also demonstrated that when subxyphoid site for drain insertion was used, the pain score was similar to the pleural integrity maintenance. Furthermore, mechanical ventilation time, incidence of atelectasis, pleural effusion and postoperative hospital stay were similar when compared the intact pleura to the pleural drain in subxyphoid position. Most likely, these outcomes resulted from the minimized chest wall trauma influenced by the change of the pleural drainage site to the subxyphoid position.

A large body of evidence demonstrates that preservation of pleural integrity during harvesting the ITA graft is associated to lower degrees of pulmonary dysfunction. The present study showed that even in the presence of pleurotomy, shifting the drain pleural insertion site to the subxyphoid position may constitute an excellent alternative technique to preserve the chest wall and decrease impairment in pulmonary function, as compared to intact pleura. This finding might be important for patients with impaired pulmonary function like chronic obstructive pulmonary disease and also those who are at risk of postoperative respiratory complication. Additionally, this information could be of relevant interest when both pleura are entered and bilateral drainage of pleural cavities becomes necessary.

Therefore, the original findings of this study suggest that in the presence of pleurotomy, placement of pleural drain in subxyphoid position may represent a clear advantage to decrease trauma to the chest wall and minimize pulmonary dysfunction in the early postoperative period.

Although the impact of comparative direct or indirect costs between groups has not been assessed, our results showed no significant difference in clinical outcomes, such as, mechanical ventilation time, incidence of the atelectasis and pleural effusion and hospital stay.

## Conclusion

The subxyphoid insertion of pleural drain provides similar effects to preserved pleural integrity in pulmonary function, thoracic pain, and clinical outcomes after OPCAB. Therefore, our results support the hypothesis that, once pleural cavities are incidentally or purposely opened during LITA graft dissection, subxyphoid placement of the pleural drain is recommended.

## List of abbreviations

CABG: Coronary artery bypass grafting; FEV_1_: Forced expiratory volume in 1 second; FVC: Forced vital capacity; ITA: Internal thoracic artery; LITA: Left internal thoracic artery; OPCAB: Off-pump coronary artery bypass; PaCO_2_: Partial pressure of carbon dioxide; PaO_2_: Partial pressure of arterial oxygen; POD: Postoperative day.

## Competing interests

The authors declare that they have no competing interests.

## Authors' contributions

SG: Conceived the study and designed the study, analysis and interpretation of data and coordination and wrote the manuscript. DWB: Participated in the design of the study, performed the statistical analysis and helped to draft the final manuscript. SMF: Participated in the sequence alignment, have been involved in drafting the manuscript, revising it critically for important intellectual content. RFF: Participated in the design of the study and performed the statistical analysis. KT: Collection of data, analysis and interpretation of data. AAC: Participated in the design, analysis and interpretation of data. WJG: Conceived the study, coordination and revising it critically for important intellectual content. All authors read and approved the final manuscript.
